# Addition of n-butyl cyanoacrylate to classic transarterial chemoembolization may improve the radiological response in patients with hepatocellular carcinoma

**DOI:** 10.6061/clinics/2015(12)04

**Published:** 2015-12

**Authors:** Lucas Moretti Monsignore, Jorge Elias-Junior, Valdair Francisco Muglia, Andreza Correa Teixeira, Enio David Mente, Ana de Lourdes Candolo Martinelli, Daniel Giansante Abud

**Affiliations:** I Universidade de São Paulo, Faculdade de Medicina de Ribeirão Preto, Departamento de Clínica Médica, Divisão de Radiologia, Ribeirão Preto/SP, Brazil.; II Universidade de São Paulo, Faculdade de Medicina de Ribeirão Preto, Departamento de Cirurgia e Anatomia, Divisão de Cirurgia Digestiva, Ribeirão Preto/SP, Brazil.; III Universidade de São Paulo, Faculdade de Medicina de Ribeirão Preto, Departamento de Clínica Médica, Divisão de Gastroenterologia, Ribeirão Preto/SP, Brazil.

**Keywords:** Liver, Hepatocellular carcinoma, Therapeutic chemoembolization, Enbucrilate

## Abstract

**OBJECTIVE:**

Transarterial chemoembolization is the treatment of choice for intermediate-stage hepatocellular carcinoma. However, there are no clear data supporting transarterial chemoembolization *vs*. transarterial embolization or regarding the best chemotherapeutic agent, which may suggest a preponderant role of ischemia over chemotherapeutic action. This study sought to evaluate the radiological response and outcome of transarterial chemoembolization modified by n-butyl cyanoacrylate addition compared to conventional transarterial chemoembolization in hepatocellular carcinoma patients.

**MATERIALS AND METHODS:**

A retrospective review identified forty-seven patients who underwent modified chemoembolization and thirty-three who underwent conventional chemoembolization between June 2006 and December 2011. The radiological response was reassessed using the modified Response Evaluation Criteria in Solid Tumors. The sustained complete response, time to progression and overall survival rates were also analyzed.

**RESULTS:**

Complete response rates were significantly higher in patients who had undergone modified chemoembolization compared to those who had undergone conventional treatment (61.7% and 24.3%, respectively; *p*<0.001). The rate of sustained complete response was significantly higher in the modified chemoembolization group compared to the conventional chemoembolization group (median of 236 and 37 days, respectively; *p*<0.001). Time to progression was significantly higher in the modified chemoembolization group compared to the conventional chemoembolization group (median of 424 and 201 days, respectively; *p*=0.042). Overall survival rates revealed no difference between patients who received modified chemoembolization and conventional chemoembolization (median of 483 and 399 days, respectively; *p*=0.316).

**CONCLUSION:**

Transarterial chemoembolization modified by n-butyl cyanoacrylate addition was superior to conventional transarterial chemoembolization in terms of the radiological response in the first imaging control. Although the sustained complete response and time to progression rates were higher for the modified chemoembolization group, no differences in overall survival rates were observed.

## INTRODUCTION

Transarterial chemoembolization (TACE) is the treatment of choice for intermediate-stage hepatocellular carcinoma (HCC) 1, although this approach is also used for less advanced cases as a bridge therapy to liver transplantation 2. However, there are no clear data supporting TACE over transarterial embolization (TAE) and there is no significant difference in survival rates between patients who receive TAE or TACE 3,4. Additionally, no chemotherapeutic agent has been shown to be better than others in TACE procedures 3. Therefore, the ischemic effect of TACE might be dominant over the chemotherapeutic effect.

N-butyl cyanoacrylate (NBCA) is a preferred embolic material for hemorrhage treatment when feasible, due to its fast preparation and delivery as well as its independence from clotting factors to successfully stop bleeding 5. In our institution, we observed that TACE cases that presented with intratumoral bleeding during polyvinyl alcohol microsphere deployment, which were promptly treated with NBCA, presented better radiological responses compared to those that underwent conventional TACE (cTACE). For these reasons, in mid-2010, NBCA was regularly used at the end of every TACE procedure to definitively exclude the embolized arterial branch and to theoretically potentiate the ischemic effect of TACE, a procedure that we termed “TACE modified by NBCA addition” (NBCA-TACE).

Some authors have used NBCA for HCC treatment either alone or associated with TAE, but to the best of our knowledge, no studies have compared the treatment group to a control group or analyzed the radiological response of treated patients 6–8.

The aim of this study was to analyze the radiological response, sustained complete response (SCR), time to progression (TTP) and overall survival (OS) rates in patients with HCC who had undergone NBCA-TACE compared with cTACE.

## MATERIALS AND METHODS

### Patient selection

This retrospective study included consecutive patients who had undergone cTACE or NBCA-TACE between 2006 and 2011 with a minimum of 2 years of follow-up and who met all of the inclusion and exclusion criteria. The study was approved by the local ethics committee.

The inclusion criteria were as follows: diagnosis of HCC according to the American Association for the Study of Liver Diseases guidelines 1 and treatment with cTACE or NBCA-TACE between 2006 and 2011 at our institution. The exclusion criteria were as follows: diagnosis of any tumor other than HCC; failure to proceed in cTACE or NBCA-TACE due to high-flow arteriovenous fistula; unavailability of multidetector computed tomography (MDCT) or magnetic resonance imaging (MRI) for review and reassessment before the procedure; and refusal of patients or family members to have their data included in this study.

### Clinical information

Clinical data were collected from medical records, except the following information was collected from medical records or through telephone calls to patients or family: performance of curative procedures (ablative methods, surgical resection or liver transplantation) and its realization date; date of death for deceased patients.

### Pre-TACE imaging evaluation, TACE procedure evaluation and post-TACE imaging evaluation

MDCTs or MRIs that were performed at the time of diagnosis were reassessed by two dedicated abdominal radiologists (20 years and 18 years of experience) and the following information was collected: sum of the largest lesion diameters; rating the largest lesion in accordance with its largest diameter (less than 2 cm, 2 to 3 cm, 3 to 5 cm, greater than 5 cm); classification according to number and appearance (uninodular, multinodular or infiltrative); presence of portal vein thrombosis; and liver segments with tumors.

TACE patients were reassessed by two experienced interventional radiologists (12 and 8 years of experience) and the following information was collected: anatomical hepatic artery variations according to the classification of Michels 9; cTACE or NBCA-TACE; presence of intratumoral portal arteriovenous fistula; and whether technical success was achieved.

All of the MDCTs or MRIs performed after TACE were reassessed and classified according to the modified response evaluation criteria in solid tumors (mRECIST) 10 in terms of complete response (CR), partial response (PR), stable disease (SD) and progressive disease (PD). CR was defined as the disappearance of any intratumoral arterial enhancement in all target lesions; PR was assigned in cases with at least a 30% decrease in the sum of diameters of viable target lesions (arterial enhancing lesions), taking as reference the baseline sum of the diameters of target lesions; SD was defined as any case that did not qualify for either PR or PD; and PD was assigned when there was an increase of at least 20% in the sum of the diameters of viable (enhancing) target lesions, taking as reference the smallest sum of the diameters of viable target lesions recorded since treatment started. These results were used to evaluate the radiological response, SCR, and TTP rates.

### Baseline data

Baseline data from the groups are listed in Table 1.

### cTACE and NBCA-TACE standardization

In our Institution, all TACE procedures are performed exclusively by the Interventional Radiology team in a room dedicated to angiography, usually under local anesthesia.

Interventional procedures were performed using the femoral approach (11-cm-long 5F sheath introducer) with selective angiography of the aorta, celiac trunk, common hepatic artery and superior mesentery artery for tumor feeder characterization along with a delay phase for splenoportography using a 5F diagnostic angiography catheter.

The diagnostic angiography catheter used for selective hepatic artery angiography was irrigated with saline, which involved a microcatheter (Renegade 18, Boston Scientific, Natick, Massachusetts, USA) with an inner lumen diameter of 0.021” and a 0.014” micro-guidewire (ChoICE, Boston Scientific) being navigated coaxially until they reach the supplying arteries of the tumor.

**Figure 1:**
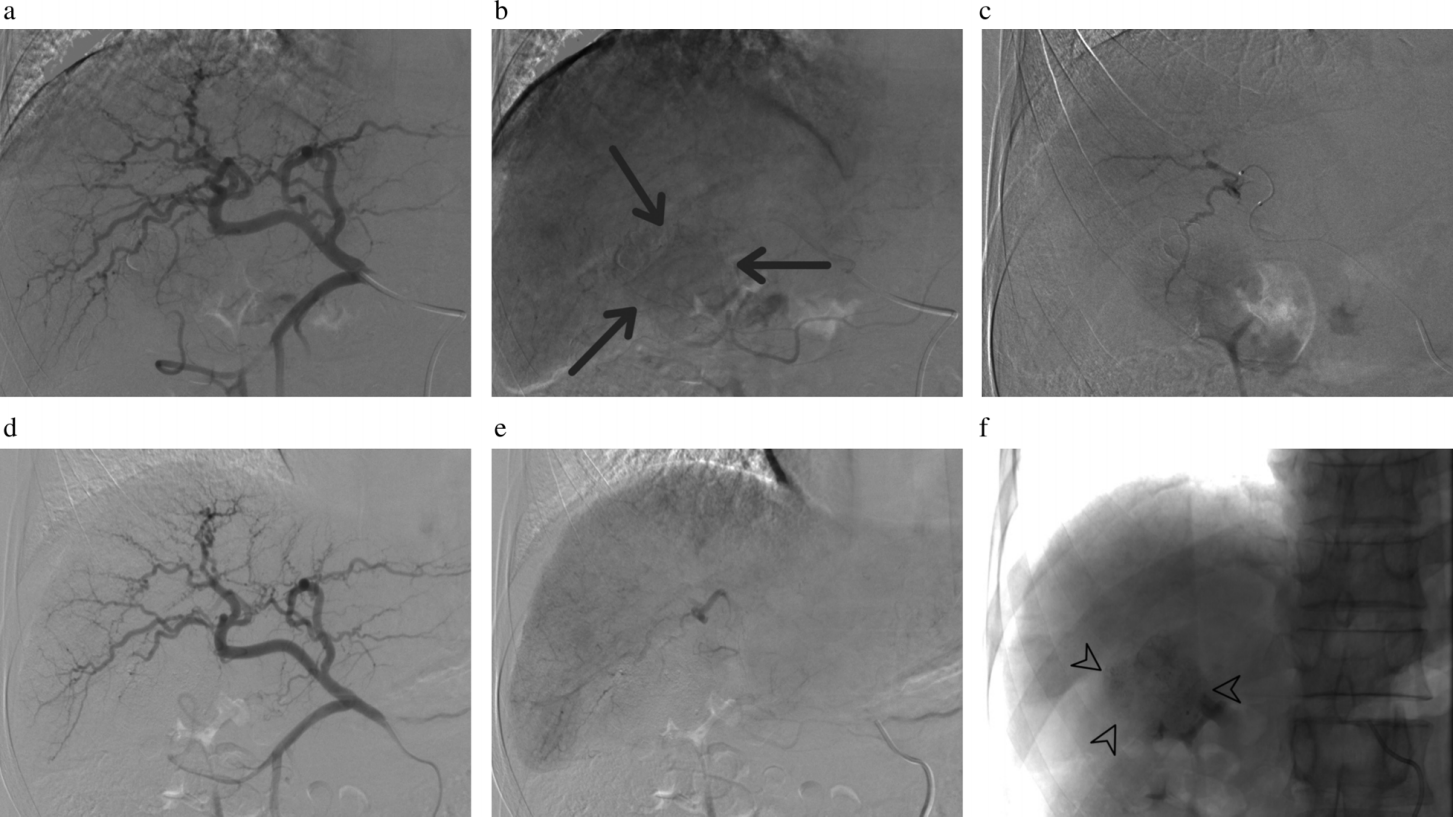
**a-f:** cTACE standardization. Selective angiography of the common hepatic artery (a, b) depicts a hypervascular lesion nourished by distal branches of the hepatic artery (arrows). Microcatheters reached the supplying arteries of the tumor (c). No staining of the tumor was seen in the final angiography (d, e). The procedure was considered to be successful. The tumor retained Lipiodol and became radiopaque (f, arrowheads).

An emulsion containing a maximum of 10 mg mitomycin diluted in distilled water to a total volume of 20 ml plus 2 ml iodized oil (Lipiodol, Laboratoire André Guerbet, Aulnay-sous-Bois, France) was applied through the microcatheter followed by the application of 300 to 500 micrometer polyvinyl alcohol microspheres (BeadBlock, Biocompatibles, UK) until stagnation of blood flow and a lack of tumor staining was achieved. The cTACE was deemed successful when there was no tumor staining (Figure 1).

**Figure 2:**
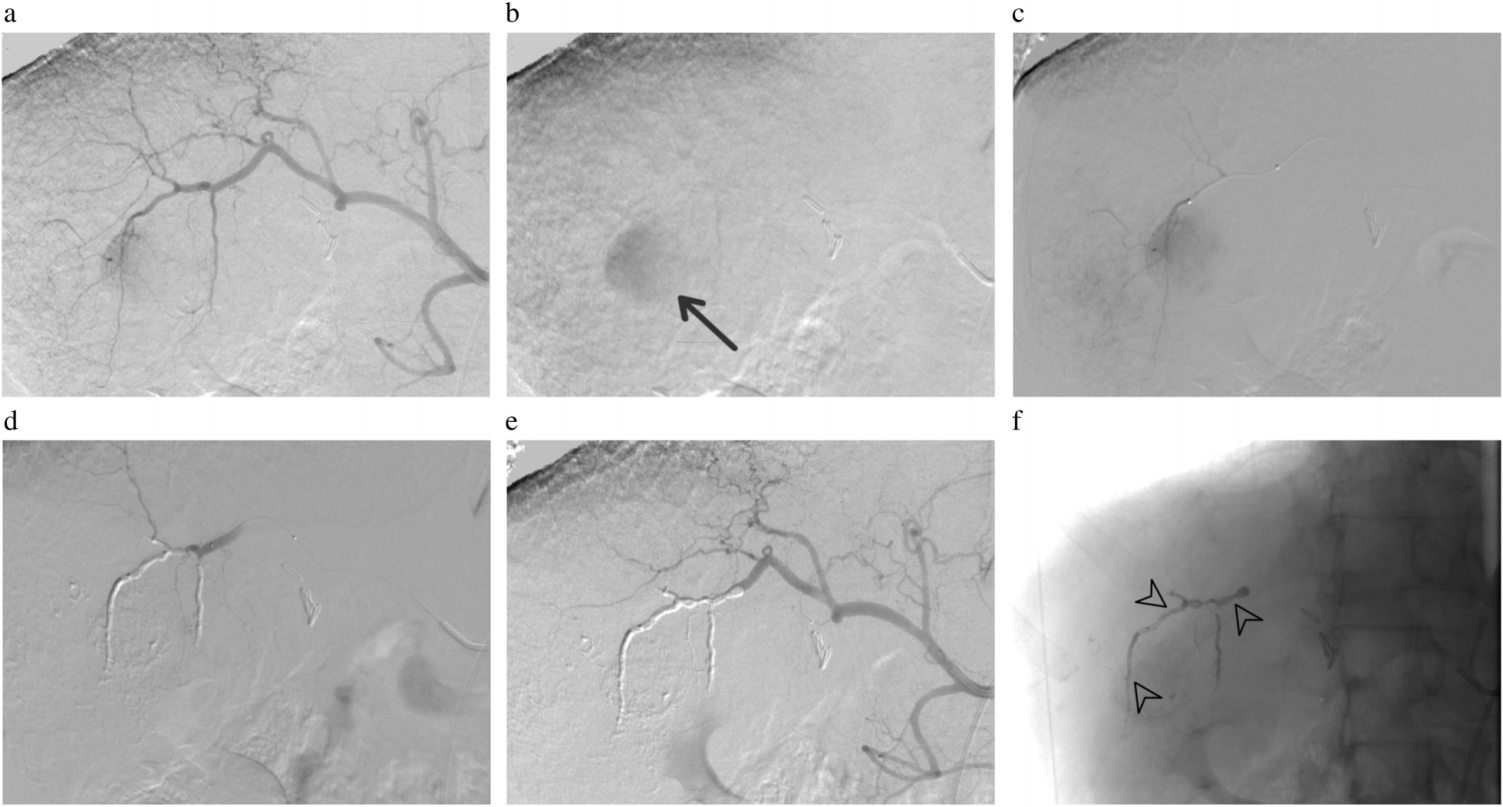
**a-f:** NBCA-TACE standardization. Selective angiography of the common hepatic artery (a, b) depicts a hypervascular lesion nourished by distal branches of the hepatic artery (arrows). Microcatheters reached the supplying arteries of the tumor (c). After the third microcatheterization, with two prior occlusions of smaller distal branches via the application of NBCA-Lipiodol mixture, no staining of the tumor was seen (d). Application of the NBCA-Lipiodol mixture was performed to definitively occlude that branch and final angiography showed complete occlusion of the branches directed to the tumor (e). The NBCA-Lipiodol mixture cast inside the occluded branches is shown by arrowheads (f).

In NBCA-TACE, after the steps described above, the microcatheter was flushed with 5% dextrose solution, followed by the injection of a mixture of NBCA (Histoacryl, B. Braun AG, Melsungen, Germany) and iodized oil in a 1:3 to 1:5 ratio (33%–20% NBCA), depending on the blood flow velocity and vessel caliber, until the artery was completely obstructed. NBCA-TACE was successful when all tumor feeders previously observed were excluded from the circulation (Figure 2).

After the angiographic controls, the introducer sheath was withdrawn followed by manual compression of the punctured site until hemostasis was achieved, which lasted for approximately 15 minutes.

After the procedure, the patients remained in the hospital for at least 24 hours to control symptoms, and they were then released.

### Radiological response and SCR, TTP and OS evaluation

Radiological responses were evaluated using the mRECIST 10; these modified criteria are used to assess HCC patients who have undergone locoregional or systemic treatment and have been modified from the response evaluation criteria in solid tumors (RECIST) 11. mRECIST classifies the target tumor observed on the first-month MDCT or MRI as CR, PR, SD or PD.

SCR was defined as the elapsed time since cTACE or NBCA-TACE until the first evaluated MDTC or MRI was classified different from CR according to mRECIST. Censored data included death from any cause, patients who had undergone any other HCC treatment after the first cTACE or NBCA-TACE or patients who were lost to follow-up.

TTP was defined as the elapsed time since cTACE or NBCA-TACE until the first evaluated MDTC or MRI was classified as PD according to mRECIST. Censored data included death from any cause, patients who had undergone any other HCC treatment after the first cTACE or NBCA-TACE or patients who were lost to follow-up.

OS was defined as the elapsed time from cTACE or NBCA-TACE until death. Censored data included patients who had undergone any curative treatment or patients who were lost to follow up.

### Follow-up

The follow-up protocol for patients undergoing TACE at our institution included obtaining MRI or MDCT controls one, three and six months after the procedure and semiannually thereafter. In the case of PR or SD, new TACE was proposed according to the patient's clinical condition. If the imaging method used for the control defined PD, a multidisciplinary team decided whether to perform a new TACE, to use sorafenib or to provide the best supportive care. In our institution, sorafenib and TACE have not been used concurrently.

For patients who fit the Milan criteria and achieved downstaging, liver transplantation was performed in accordance to our health system rules.

### Statistical analysis

Continuous data were expressed as the mean ± standard deviation and analyzed with Student’s t test. The Mann-Whitney U test was used for ranked data and Fisher’s exact test was used to assess the association between two qualitative variables. The Kaplan-Meier method using the Mantel-Cox test was employed for calculating SCR, TTP and OS rates. A *p*-value less than 0.05 was considered statistically significant. Statistical analysis was performed with IBM ® SPSS ® Statistics program, version 20 for Mac.

## RESULTS

Eighty patients met the inclusion criteria and were enrolled in the study. Of these, forty-seven patients underwent NBCA-TACE and thirty-three underwent cTACE. There were no significant differences in the baseline data (*p*>0.05) except for hepatitis C virus infection, which was more frequent in the NBCA-TACE group (*p*=0.005) and MDCT, which was more frequent for the cTACE group than for the NBCA-TACE group in the first imaging control (*p*<0.001).

### Radiological response

Of all the patients, four did not have their first imaging control analyzed and classified according mRECIST. These patients included one patient from the cTACE group (patient passed away before the first imaging control) and three from the NBCA-TACE group (one underwent a liver transplantation a few days after the procedure and two patients did not undergo dynamic MDCT or MRI; one due to clinical contraindication and the other due to patient refusal, thus not amenable to analysis). For the cTACE group, the response analysis of the target lesion according to the mRECIST criteria revealed 24.3% of patients with CR, 54.6% with PR, 12.1% with SD, 6.1% with PD and 3.0% who were not amenable for analysis. For the NBCA-TACE group, this distribution was 61.7% with CR, 23.4% with PR, 4.3% with SD, 4.3% with PD and 6.4% who were not amenable for analysis (*p*<0.001). These data are summarized in Table 2.

### Sustained complete response

**Figure 3:**
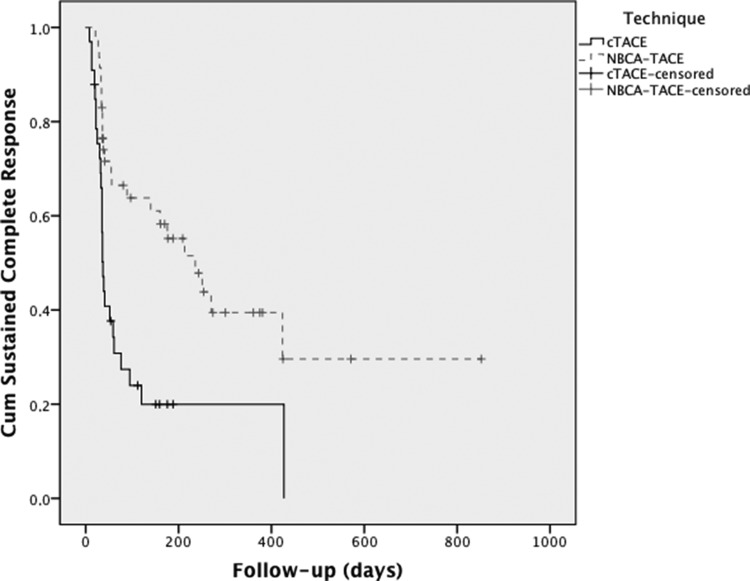
Kaplan-Meier curve of SCR of patients undergoing cTACE and NBCA-TACE.

The NBCA-TACE group demonstrated a significantly longer SCR compared to the cTACE group. The NBCA-TACE group had an estimated mean and median SCR of 360.65 and 236 days, respectively, while the cTACE group had an estimated mean and median of 120.8 and 36 days, respectively, with a significant difference in Kaplan-Meier curves according to the Mantel-Cox test (*p*<0.001) (Figure 3).

### Time to progression

**Figure 4:**
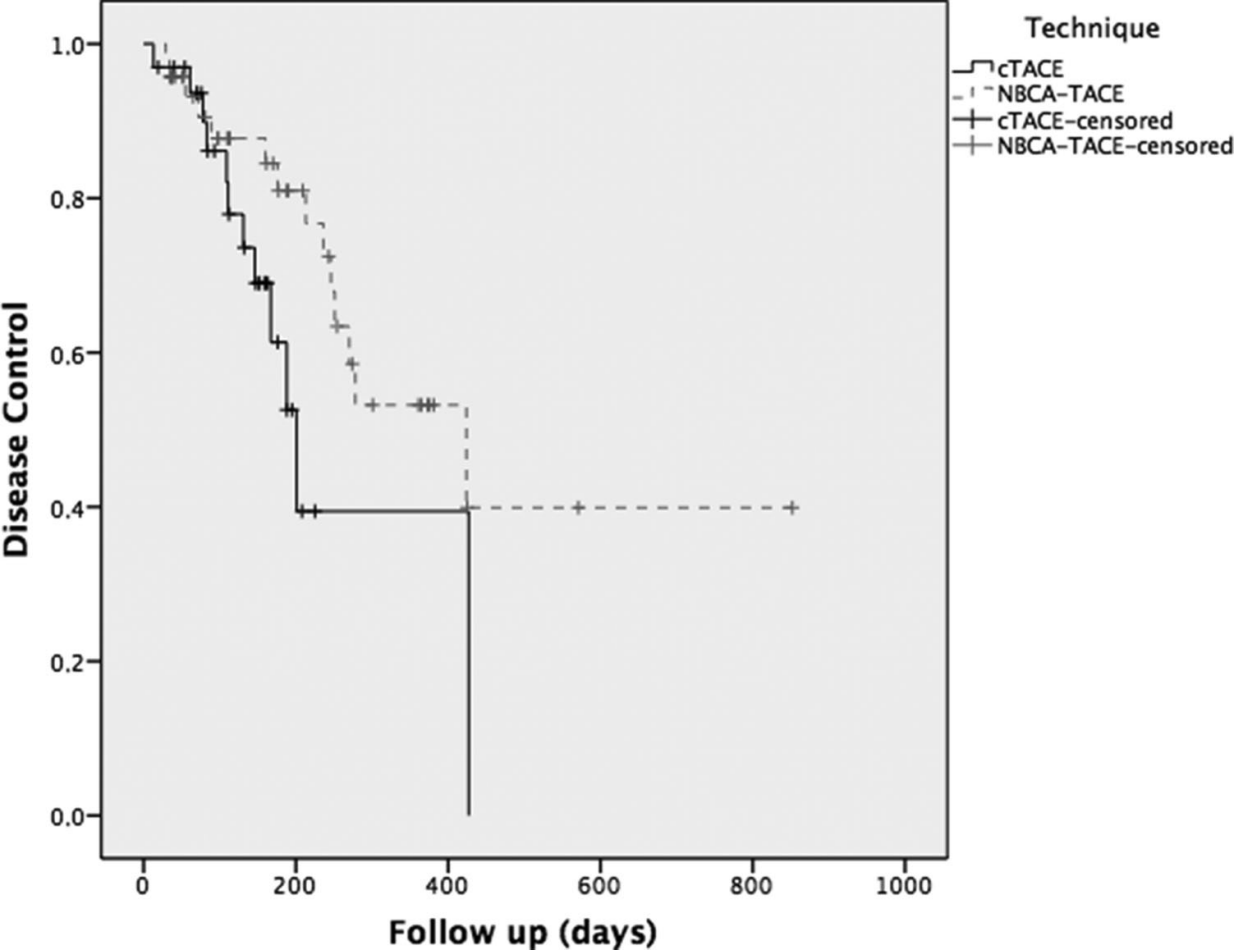
Kaplan-Meier curve of TTP of patients undergoing cTACE and NBCA-TACE.

The NBCA-TACE group demonstrated a significantly longer TTP compared to the cTACE group. The NBCA-TACE group had an estimated mean and median TTP of 484.5 and 424 days, respectively, while the cTACE group had an estimated mean and median of 253.9 and 201 days, respectively, with a significant difference in Kaplan-Meier curves according to the Mantel-Cox test (*p*=0.042) (Figure 4).

### Overall survival

**Figure 5:**
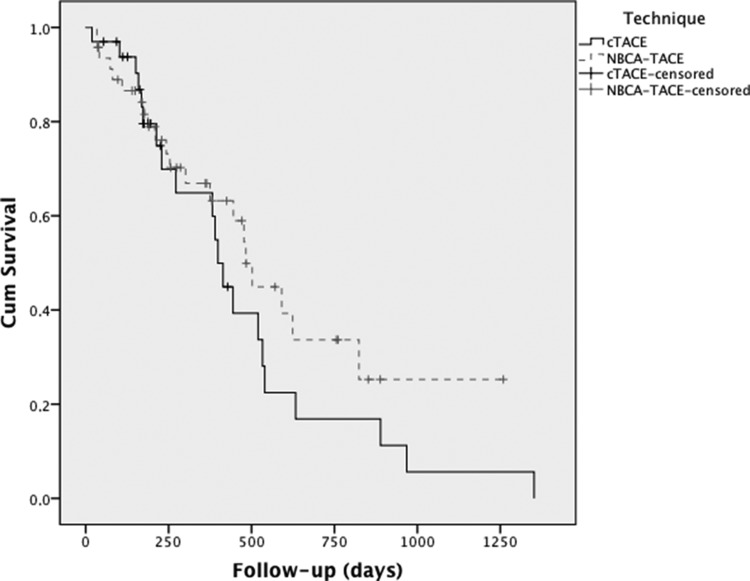
Kaplan-Meier curve of OS of patients undergoing cTACE and NBCA-TACE.

The mean and median estimated OS rates of the cTACE group were 470.49 and 399 days, respectively, while in the NBCA-TACE group, these values were 613.97 and 502 days, respectively. There were no significant differences between groups according to the Mantel-Cox test (*p*=0.316) (Figure 5).

### Complications and post-procedure side effects

There were no differences between the rates of post-embolization syndrome symptoms in both the cTACE and NBCA-TACE groups; abdominal pain was the major presented symptom followed by nausea, fever and vomiting (Table 3).

## DISCUSSION

TACE is employed worldwide for the treatment of intermediate HCC, which is characterized by no severe compromise in liver function (Child A or B), multinodular disease and no signs of vascular invasion or extra hepatic spread 12. In addition, TACE has been used not only in patients with early-stage HCC but also as a bridge to transplant 13 and in advanced disease stages, in association with other therapies 14.

The use of different chemotherapeutic agents and diverse embolic materials worldwide makes cTACE a non-standardized procedure. Although the introduction of doxorubicin-eluting beads in TACE (DEBDOX-TACE) standardized this procedure, no clear benefits in terms of tumor response or survival have been observed 15,16.

Despite its inclusion in the algorithm of HCC treatment 1,17, TACE has not yet been demonstrated to be superior over TAE for survival 3,4. The lack of evidence showing superiority of one chemotherapeutic agent over the other in TACE procedures 3, as well as the absence of superiority of TACE over TAE, may suggest a preponderance of the ischemic effect over the chemotherapeutic effect. Approximately 90% of the HCC blood supply originates from the hepatic artery, while only 10% is from the portal vein, although the normal liver depends on approximately 25% of nutrients coming through the hepatic artery and 75% through the portal vein 18, which could explain this possible preponderance of the ischemic effect of TACE. Some authors suggest that further studies are needed to evaluate the impact of maximization of ischemia in TACE on HCC patients 3.

To our knowledge, there are only three reports on the use of NBCA in TAE in HCC patients using different techniques as an alternative to cTACE in the English literature 6–8. However, there was no cTACE group as a control in any of these studies and none of the studies assessed tumor response using imaging methods. Berghammer P et al. 6 performed a retrospective analysis to evaluate the use of a Lipiodol-NBCA mixture alone in 16 patients with unresectable HCC and observed few side effects with no survival benefit (median survival of 8.5 months). Loewe C et al. 7 retrospectively studied 36 patients with unresectable HCC embolized with a Lipiodol-NBCA mixture alone and observed a median survival of 26 months, which was potentially prolonged compared to reports of other embolization techniques in the literature. In a retrospective study including 46 patients with unresectable HCC, Rand T et al. 8 studied embolization with Trisacryl microspheres followed by a Lipiodol-NBCA mixture to definitively occlude the embolized territory; however, this study did not use any chemotherapeutic agent. These authors observed a median survival of 666 days after diagnosis, similar to other techniques described in the literature.

Regarding the tumor response assessed by mRECIST, three studies were found in the literature that compared cTACE to DEBDOX-TACE. In 2011, Song MJ et al. 19 published a retrospective controlled study with 40 HCC patients who had undergone cTACE using doxorubicin as a chemotherapeutic agent or DEBDOX-TACE. These authors observed a significant difference in the objective response (CR plus PR) in imaging controls one month after the procedures (*p*=0.001). The authors found the following distribution for CR, PR and SD: cTACE 20%, 10% and 70%; and DEBDOX-TACE 35%, 50% and 15%, respectively. In 2012, Song MJ et al. 20 published a retrospective cohort including 129 patients with HCC who had undergone cTACE using doxorubicin as a chemotherapeutic agent or DEBDOX-TACE. The imaging control that was assessed for mRECIST evaluation was taken 3 months after the procedure. The DEBDOX-TACE group presented as 55.0% CR, 26.6% PR, 15.0% SD and 3.4% PD, while the cTACE group presented as 23.1% CR, 26.3% PR, 30.4% SD and 20.2% PD. The difference between groups was significant at *p*<0.001. Malagari K et al. 21 performed a prospective study including HCC patients who were classified as BCLC A with unresectable lesions or lesions that were not amenable for percutaneous ablation and were classified as BCLC B. All of the patients were scheduled for three sessions of DEBDOX-TACE unless CR was achieved in fewer sessions. Imaging controls were performed 30 days after the last procedure and assessed by mRECIST. These authors observed the following results in the evaluation of target lesions: 22.2% CR, 46.6% PR, 22.2% SD and 8.8% PD.

**Figure 6:**
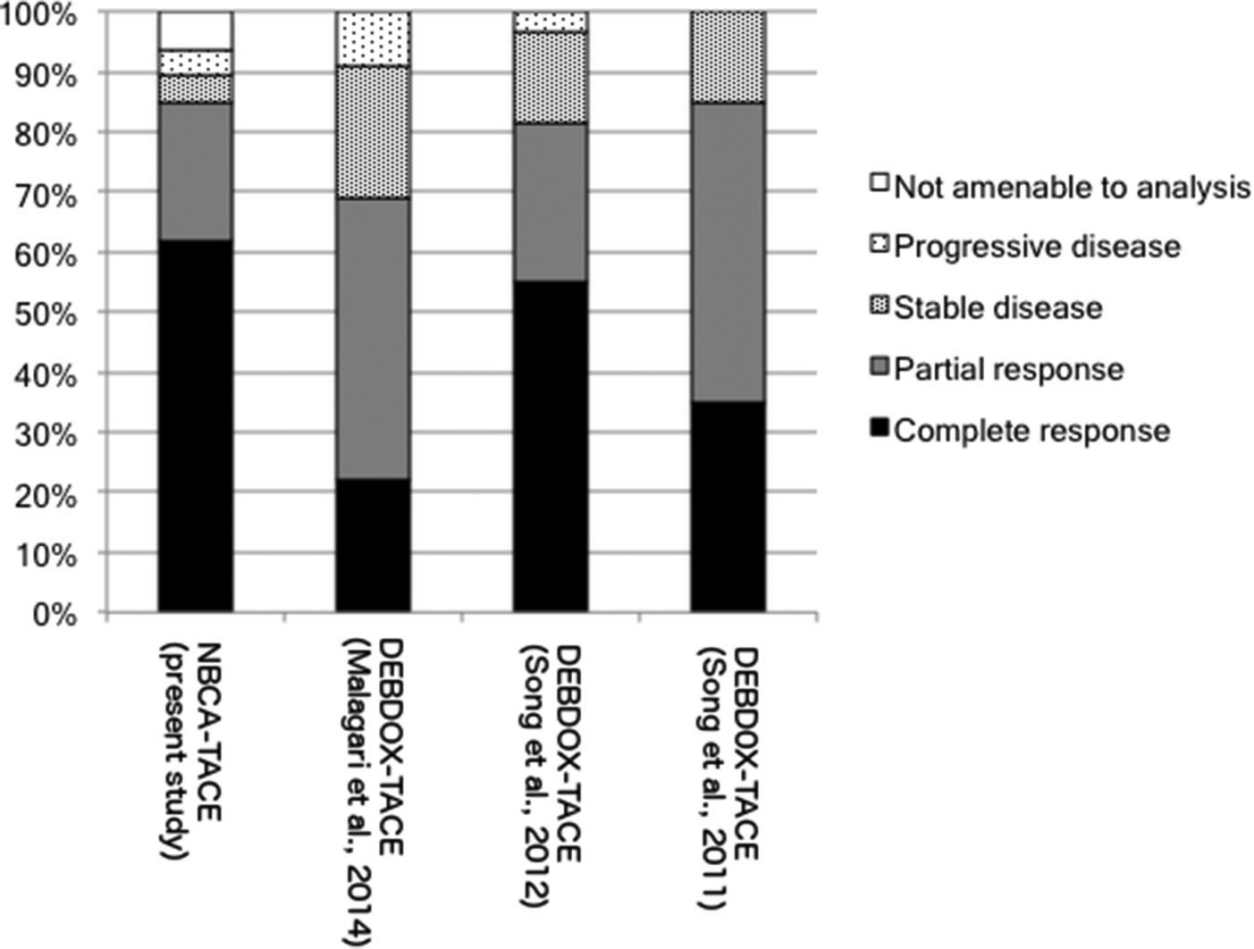
Radiological responses assessed by mRECIST found in the present study compared to other studies in the literature.

In the present study, the radiological response assessed by mRECIST for the NBCA-TACE group was better in comparison to any other study, with 61.7% CR, 23.4% PR and 4.3% SD; these results were also superior to those observed for the cTACE group, with 24.3% CR, 54.6% PR and 12.1% SD (Figure 6).

Despite the fact that there was no increase in the OS rate of the NBCA-TACE group compared with the cTACE group or even in the use of NBCA for HCC embolization in other studies 6–8, we observed a significant increase in the SCR and TTP rates of the NBCA-TACE group compared to the cTACE group, which may lengthen the waiting time for liver transplantation within the Milan criteria. This finding also demonstrates the superiority of NBCA-TACE in terms of the radiological responses and may consequently result in higher down-staging rates in selected HCC patients.

Our study has several limitations. First, this was a retrospective study examining preliminary results with a small number of patients who had been divided into two heterogeneous but comparable groups. Second, this study was performed by experienced interventional radiologists in a single center, which may have biased the results. Third, as NBCA-TACE is a new procedure with an embolic material that is different from what has been previously used, there may have been a learning curve that could have distorted the results, although this technique is already routinely used by staff in other environments.

In conclusion, NBCA-TACE was superior to cTACE in terms of the radiological response and SCR and TTP in HCC patients, with no difference regarding OS in the studied groups. Future prospective randomized controlled studies with larger cohorts are required to confirm our findings.

## AUTHOR CONTRIBUTIONS

Monsignore LM conceived the study and participated in its design, coordination and data collection, as well as drafted the manuscript. Elias-Junior J and Muglia VF participated in the study design, data collection and imaging review. Teixeira AC, Mente ED and Martinelli AL participated in the study design and data collection. Abud DG conceived the study, participated in its design and coordination, and helped drafting the manuscript.

## Figures and Tables

**Table 1:** Baseline data of the groups.

	cTACE	NBCA-TACE	*p*-value
Number of patients	33	47	
Male (%)	26 (78.79)	41 (87.23)	0.365
Age (mean ± sd)	56.4 ± 10.3	58.3 ± 8.9	0.727
Etiology of cirrhosis			
Hepatitis B (%)	4 (12.12)	7 (14.89)	1.000
Hepatitis C (%)	13 (39.39)	34 (72.34)	0.005
Alcohol (%)	22 (66.67)	34 (72.34)	0.626
Other causes (%)	1 (3.03)	0 (0.00)	0.412
Aim of TACE			
Maintenance in Milan’s criteria (%)	13 (39.39)	24 (51.06)	0.212
Downstaging (%)	13 (39.39)	10 (21.28)	
Palliative (%)	7 (21.21)	13 (27.66)	
Performance of any curative treatment (%)	10 (30.30)	19 (40.43)	0.479
Performance of new cTACE or NBCA-TACE (%)	16 (48.49)	17 (36.17)	0.192
cTACE or NBCA-TACE per patient (mean ± sd)	1.67 ± 0.89	1.55 ± 0.90	0.580
Laboratory tests			
Total bilirubin, in mg/dL (mean ± sd)	1.93 ± 1.52	1.74 ± 0.88	0.117
Albumin, in g/L (mean ± sd)	3.68 ± 0.43	3.53 ± 0.75	0.122
INR (mean ± sd)	1.24 ± 0.19	1.22 ± 0.15	0.163
Alpha-fetoprotein	291.6 ± 731.0	337.4 ± 859.8	0.606
Serum creatinine, in mg/dL (mean ± sd)	0.99 ± 0.32	0.91 ± 0.25	0.085
Serum sodium, in mmol/L (mean ± sd)	139.6 ± 3.7	140,2 ± 3.0	0.422
Sum of the largest diameter of all lesions identified on the basal MDCT or MRI, in cm (mean ± sd)	6.6 ± 3.8	5.3 ± 2.6	0.185
Largest diameter of the biggest lesion on the basal MDCT or MRI, in cm			
< 2.0 cm (%)	2 (6.06)	3 (6.38)	0.207
> 2.0 cm < 3.0 cm (%)	5 (15.15)	14 (29.79)	
> 3.0 cm < 5.0 cm (%)	13 (39.39)	16 (34.04)	
> 5.0 cm (%)	13 (39.39)	14 (29.79)	
Classification in number and aspect			
Uninodular (%)	20 (60.61)	31 (65.96)	0.671
Multinodular (%)	12 (36.36)	14 (29.79)	
Infiltrative (%)	1 (3.03)	2 (4.26)	
Involved hepatic segments			
I (%)	1 (3.03)	0 (0.00)	0.412
II (%)	4 (12.12)	7 (14.89)	1.000
III (%)	5 (15.15)	7 (14.89)	1.000
IVa (%)	10 (30.30)	13 (27.66)	0.807
IVb (%)	6 (18.18)	5 (10.64)	0.347
V (%)	9 (27.27)	13 (27.66)	1.000
VI (%)	12 (36.36)	20 (42.55)	0.647
VII (%)	11 (33.33)	14 (29.79)	0.809
VIII (%)	20 (60.61)	23 (48.94)	0.365
Child-Turcotte-Pugh			
A (%)	15 (45.45)	26 (55.32)	0.452
B (%)	18 (54.55)	20 (42.55)	
C (%)	0 (0.00)	1 (2.13)	
BCLC			
0 (%)	0 (0.00)	0 (0.00)	0.355
A (%)	19 (57.58)	31 (65.96)	
B (%)	11 (33.33)	15 (31.92)	
C (%)	2 (6.06)	0 (0.00)	
D (%)	1 (3.03)	1 (2.13)	
MELD (mean ± sd)	11.55 ± 3.05	10.96 ± 2.77	0.565
Extension of embolization (% of estimated liver volume)			
0 to 25 (%)	8 (24.24)	13 (27.66)	0.294
25 to 50 (%)	17 (51.52)	29 (61.70)	
50 to 75 (%)	8 (24.24)	4 (8.51)	
75 to 100 (%)	0 (0.00)	1 (2.13)	
Technical success (%)	30 (90.91)	42 (89.36)	1.000

sd: standard deviation.

**Table 2:** Findings in the first imaging control post TACE.

	cTACE	NBCA-TACE	*p*-value
Type of exam in the first control post TACE			
MDCT (%)	13 (39.39)	3 (6.38)	< 0.001
MRI (%)	19 (57.58)	43 (91.49)	
Unperformed (%)	1 (3.03)	1 (2.13)	
Elapsed time between TACE and first control exam, in days (mean ± sd)	41.72 ± 22.52	38.63 ± 20.89	0.367
mRECIST classification of target lesion in first control exam			
CR (%)	8 (24.24)	29 (61.70)	0.001
PR (%)	18 (54.55)	11 (23.40)	
SD (%)	4 (12.12)	2 (4.26)	
PD (%)	2 (6.06)	2 (4.26)	
Impossible to evaluate (%)	1 (3.03)	3 (6.38)	

sd: standard deviation.

**Table 3:** Post embolization symptoms and complications.

	cTACE	NBCATACE	*p*-value
Nausea (%)	2 (6.06)	4 (8.51)	1.000
Vomiting (%)	1 (3.03)	1 (2.13)	1.000
Abdominal pain or discomfort (%)	8 (24.24)	17 (36.17)	0.330
Fever (%)	3 (9.09)	1 (2.13)	0.301
Other symptoms or complications (%)	3 (9.09)	0 (0.00)	0.066
